# Serum uric acid and arterial hypertension—Data from Sephar III survey

**DOI:** 10.1371/journal.pone.0199865

**Published:** 2018-07-02

**Authors:** Roxana Buzas, Oana-Florentina Tautu, Maria Dorobantu, Vlad Ivan, Daniel Lighezan

**Affiliations:** 1 Internal Medicine Department, “Victor Babes” University of Medicine and Pharmacy, Timisoara, Romania; 2 Cardio-Thoracic Pathology Department, “Carol Davila” University of Medicine and Pharmacy, Bucharest, Romania; The University of Tokyo, JAPAN

## Abstract

**Objectives:**

This paper aims to evaluate the association between serum uric acid (SUA) levels, arterial hypertension (HT) prevalence, blood pressure values control, kidney function and intima media thickness (IMT), as a surrogate marker of early atherosclerosis, in a representative group of Romanian adult population.

**Materials and methods:**

The study sample consists in 1920 adults included in SEPHAR III (**S**tudy for the **E**valuation of **P**revalence of **H**ypertension and c**A**rdiovascular **R**isk in Romania) survey (mean age 48.63 years, 52.76% females) collecting data for SUA levels, blood pressure (BP) measurements, kidney function by estimated glomerular filtration rate (eGFR) and carotid IMT. SUA levels between 2,40–5,70mg/dl in females and 3,40–7,00mg/dl in males respectively were considered normal. HT and HT control were defined according to the current guidelines. IMT evaluation was assessed by B-mode Doppler ultrasound evaluation.

**Results:**

Hypertensive subjects had significantly higher values of SUA compared with normotensive subjects, hypertensive patients were 1.713 times more likely to have higher values of SUA. Among treated hypertensive patients, those without optimal BP control had significantly higher SUA levels compared with those with optimal BP control, the presence of hyperuricemia increasing the odds of suboptimal BP control by 1.023. Hyperuricemic subjects had significantly lower eGFR values compared with normouricemic ones, on an average with 14.28ml/min/1.73m2 by Modification of Diet in Renal Disease formula (MDRD) and with 16.64ml/min/1.73m2 by Chronic Kidney Disease Epidemiology Collaboration formula (CKD-EPI), with an indirect association between SUA levels and eGFR values (rs = -0.319 / -0.347), independent of age. IMT values recorded in hyperuricemic subjects were significantly increased, on an average with 0.08mm, compared with normouricemic subjects, with a direct association between SUA levels and IMT values (rs = 0.263), independent of BP values.

**Conclusion:**

The results of our study offers support that increased SUA levels are associated with arterial hypertension and with suboptimal BP control in treated hypertensive subjects. The decline in kidney function, independent of age, and also increased IMT values as a marker of atherosclerosis, were also correlated with elevated SUA values. Hyperuricemia screening may have a role in identifying patients at risk of developing HT and lowering SUA levels may improve not only BP control in treated HT patients but also decrease total cardiovascular mortality by slowing the progression of atherosclerosis and renal failure in hypertensive patients.

## Introduction

Serum uric acid’s (SUA) involvement as an independent risk factor for cardiovascular (CV) disease is already known [1]. In recent years, SUA levels have become a novel topic of research due to the increase in the prevalence of hyperuricemia cases and the accumulated evidence that hyperuricemia increases the risk for hypertension (HT) onset and lack of optimal blood pressure (BP) control [2]. Hyperuricemia leads to the increase in BP values by stimulating oxidative stress and inflammatory mechanisms through endothelial dysfunction and proliferation of smooth muscle cells in the blood vessels and stimulation of the renin-angiotensin system. Numerous clinical studies have also shown the involvement of SUA in cardiovascular mortality in the general population, particularly in hypertensive patients with diabetes mellitus or with vascular disease [3,4].

Until SEPHAR (**S**tudy for the **E**valuation of **P**revalence of **H**ypertension and c**A**rdiovascular **R**isk in Romania) project, no representative data on SUA levels and on the prevalence of hyperuricemia were available for our adult population. SEPHAR II survey conducted in 2012 was the first national representative survey that checked SUA levels and allowed to evaluate the prevalence of hyperuricemia in our adult population and offered the data for evaluation of the link between SUA levels, arterial stiffness, renal function and total CV risk [5]. Four years later in 2014, a more complex national representative survey–SEPHAR III was conducted in which alongside SUA levels, arterial stiffness measurements and renal function evaluation by eGFR, data on intima-media thickness (IMT) were also available due to the use of B-mode Doppler ultrasound evaluation of the carotid arteries performed in this survey.

This paper aims to evaluate the association between SUA levels and HT prevalence, HT control, kidney function and IMT values, as a surrogate marker of early atherosclerosis.

### Material and methods

SEPHAR III stands out as a cross-sectional survey that provides data for the assessment of past and future trend in hypertension's prevalence, treatment and control among the adult population of Romania [6] [7]. The study included two visits, 4 days apart one from another that took part in a special ‘medical caravan’ entitled SEPHAR BUS. Of the total number of 1970 Romanian adults enrolled in SEPHAR III survey we excluded 50 subjects who had low SUA levels or were on dialysis. Therefore, the current analysis was based on a sample of 1920 adult subjects (mean age 48.63 years, 52.76% females).

During each study visit, 3 sitting BP measurements at a 1 minute interval were performed according to current guidelines (cuff adjustment for arm’s circumference, all performed at the same arm that had the highest BP value evaluated at the first study visit) using an certified automatic BP measuring device (model OMRON M6) [5,6].

HT was defined as systolic blood pressure (SBP) ≥ 140mmHg and/or diastolic blood pressure (DBP) ≥ 90mmHg at both study visits, using the arithmetic mean of the second and third BP measurement of each study visit (without taking into consideration the first BP measurement from each visit), or previously diagnosed HT under treatment during the last two weeks, regardless of BP values [6].

BP control was defined according to 2013 ESH-ESC guidelines on hypertension, as SBP < 140mmHg and DBP < 90mmHg in hypertensive subjects who were under treatment for at least 2 weeks prior, taking into account the maximum value between the two SBP/DBP values from each visit [6].

SUA evaluation was included among laboratory work-up performed during the second study visit, in fasting condition using a COBAS 6000 analyzer by enzymatic method using uricase/peroxidase reagents. The normal range for SUA levels was 2,40–5,70mg/dl in females and 3,40–7,00mg/dl in males respectively. Values above the upper limit were considered as hyperuricemia.

Kidney function was assessed by estimated glomerular filtration rate (eGFR) using both Modification of Diet in Renal Disease formula (MDRD) and Chronic Kidney Disease Epidemiology Collaboration formula (CKD-EPI).

Intima-media thickness (IMT) was measured on the distal wall of each common carotid artery at approximately 1cm prior to the carotid bulb using a linear probe (7,5 and 10 MHz) and a portable echocardiograph (model General Electric Vivid Q) with automated calculation of IMT software.

Statistical analysis was performed using IBM SPSS Statistics version 20.0 software for Windows with a significance level of 0.05. We used descriptive statistics, figures and tables to summarize our findings. Results for targeted variables were presented using descriptive statistics (mean, standard deviation, range, median, interquartile range) for continuous data, and counts with associated percentages for categorical data. Independent samples t-test was used to analyze differences in means for continuous variables, while differences between categorical variables were examined by Chi-squared test. Correlations between key data were highlighted using Spearman correlation coefficient.

The study has been conducted according to the principles expressed in the Declaration of Helsinki and with the approval of “Carol Davila” Bucharest University of Medicine and Pharmacy Ethics Committee. Written consent has been obtained from the subjects before any study procedure was performed.

## Results

**Fig 1:**
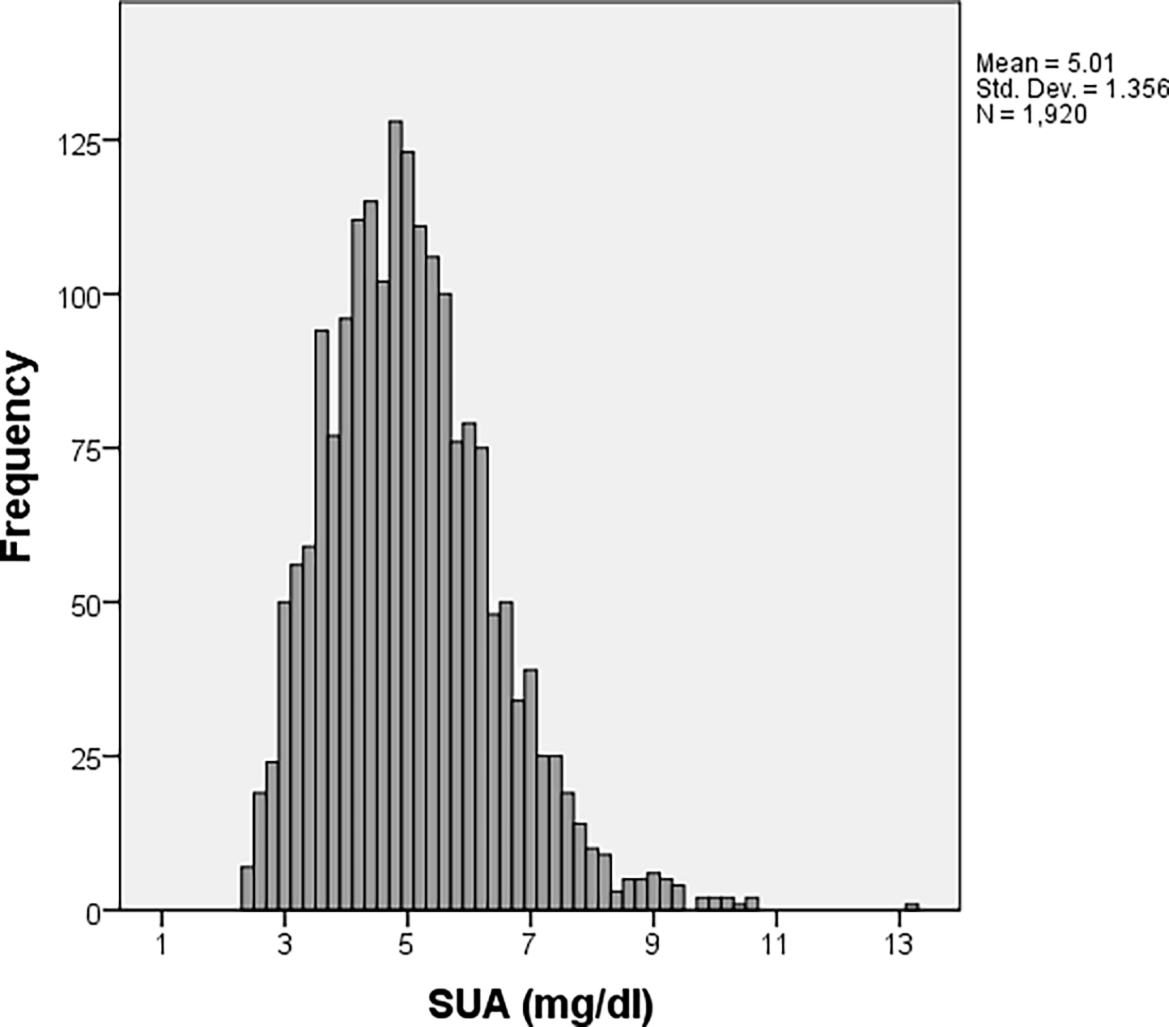
SUA levels distribution in the study population.

SUA values of the studied population ranged from 2.4 mg/dL to 13.20 mg/dL, with a median value of 4.9 mg/dL, an interquartile range of 4–5.8 mg/dL and a mean (standard deviation) value of 5.01 (1.356) mg/dL (Fig 1).

We obtained a significant statistical difference in mean values of age (Table 1). Hyperuricemia patients were older compared to normouricemia patients. No significant statistical difference was obtained among genders on the two groups of patients however patients with hyperuricemia also had a significant higher value of BMI as compared to normouricemia patients.

**Table 1:** Comparison between main baseline and anthropometric characteristics of patients with normouricemia and hyperuricemia.

	Normouricemia (N = 1694)	Hyperuricemia (N = 226)	p-value
**Age(years)**			
Mean (SD)	47.45 (17.364)	57.47 (16.382)	**<0.001**
Min; Max	18; 80	18; 80	
Median (Q1; Q3)	46 (33; 62)	61 (46; 71)	
**Gender**			
Male	799 (47.17%)	108 (47.79%)	0.861
Female	895 (52.83%)	118 (52.21%)	
**BMI (kg/m**^**2**^**)**			
Mean (SD)	27.71 (5.565)	31.97 (6.175)	**<0.001**
Min; Max	14.74; 49.59	18.56; 52.36	
Median (Q1; Q3)	27.27 (23.62; 31.07)	31.47 (27.63; 35.71)	

Significant statistical difference among the proportion of normotensive and hypertensive patients in the two groups of normouricemia and hyperuricemia could be observed. 68.58% of patients with hypertension also had hyperuricemia compared with 31.42% in the normotensive group (p<0.001) (Table 2).

**Table 2:** Comparison between arterial blood pressure groups, IMT and eGFR between patients with normouricemia and hyperuricemia.

	Normouricemia(N = 1694)	Hyperuricemia(N = 226)	p-value
**Categories for blood pressure**			
Normotensive	969 (57.20%)	71 (31.42%)	**<0.001**
Hypertensive	725 (42.80%)	155 (68.58%)	
**Hypertension–including only**			
**Hypertensive patients**			
Under control	174 (24%)	22 (14.19%)	**0.008**
Not under control	551 (76%)	133 (85.81%)	
**IMT (mm)**			
Mean (SD)	0.64 (0.151)	0.72 (0.160)	**<0.001**
Min; Max	0.34; 1.26	0.41; 1.32	
Median (Q1; Q3)	0.61 (0.52; 0.73)	0.69 (0.60; 0.83)	
**eGFR_MDRD_**			
Mean (SD)	83.43 (18.296)	69.15 (19.529)	**<0.001**
Min; Max	13.10; 230.97	4.51; 121.27	
Median (Q1; Q3)	82.22 (71.40; 94.03)	70.37 (60.60; 80.04)	
**eGFR_CKD–EPI_**			
Mean (SD)	90.69 (19.097)	74.05 (21.675)	**<0.001**
Min; Max	12.55; 150.58	4.01; 122.66	
Median (Q1; Q3)	91.03 (77.78; 104.05)	75.82 (62.49; 88.43)	

SD = Standard Deviation, Q1 = 25 percentage quartile, Q3 = 75 percentage quartile

Analyzing SUA levels by considering the presence of arterial hypertension, we obtained significantly higher values in hypertensive patients compared to those recorded in normotensive patients. The differences remained statistically significant after adjusting by age, sex and BMI (Table 3).

**Table 3:** Serum uric acid by groups of normotensive and hypertensive patients; p-values are obtained with ANCOVA test. Values are summarized as mean (standard error).

	Normotensive (N = 1040)	Hypertensive (N = 880)	p-value
**Unadjusted**	4.76 (0.041)	5.30 (0.045)	**<0.001**
**Adjusted for age**	4.83 (0.042)	5.22 (0.046)	**<0.001**
**Adjusted for gender**	4.80 (0.037)	5.26 (0.040)	**<0.001**
**Adjusted for BMI**	4.86 (0.040)	5.18 (0.044)	**<0.001**

Hypertensive subjects had significantly higher SUA levels compared to normotensive subjects, on average with 0.54 mg/dL (5.30 mg/dL vs. 4.76 mg/dL, p<0.001). In addition, SUA levels in hypertensive subjects with uncontrolled hypertension were significantly higher, on average with 0.58 mg/dL, compared to hypertensive subjects with blood pressure values under control (5.43 mg/dL vs. 4.85 mg/dL, p<0.001).

Logistic regression analysis revealed statistically significantly higher odds for hyperuricemia for increased age, increased BMI and patients with hypertension (Table 4).

**Table 4:** Multiple logistic regression analysis for risk of hyperuricemia.

	Patients with hyperuricemia n (%)*	OR (95% CI)	p-value
**Age (years)**	226 (11.77%)	1.023 (1.031; 1.033)	<0.001
**BMI (kg/m**^**2**^**)**	226 (11.77%)	1.099 (1.027; 1.127)	<0.001
**Hypertension presence**			
**Normotensive**	71 (3.70%)	1	
**Hypertensive**	155 (8.07%)	1.713 (1.241; 2.363)	0.001

Note: 95% CI = 95% confidence interval.

* Percentages based on the total number of patients (1920 patients).

The odds of hyperuricemia were higher for older patients and also for patients with higher BMI. For each additional year, the odds of hyperuricemia are higher by a factor of 1.023 (OR = 1.023, 95%CI 1.031; 1.033).

Hypertensive patients have 1.713 higher odds of hyperuricemia as compared to normotensive patients (OR = 1.713, 95%CI 1.241; 2.363).

Out of the 725 hypertensive patients only 174 had controlled arterial blood pressure values. SUA values were significantly increased in the uncontrolled hypertension group (p<0.008).

When studying IMT values, significant difference in mean value of IMT was obtained. Hyperuricemia patients had a significant higher IMT value, on average by 0.08mm, compared to normouricemia patients (0.69mm vs 0.6mm1, p<0.001).

Analyzing IMT values by groups of normouricemia and hyperuricemia patients, we noticed that IMT values recorded in the hyperuricemia group were statistically significantly higher than those recorded in the normouricemia group. The differences remained statistically significant after adjusting by sex and BMI. However, when adjusting the analysis by age the difference was statistically similar.

**Fig 2:**
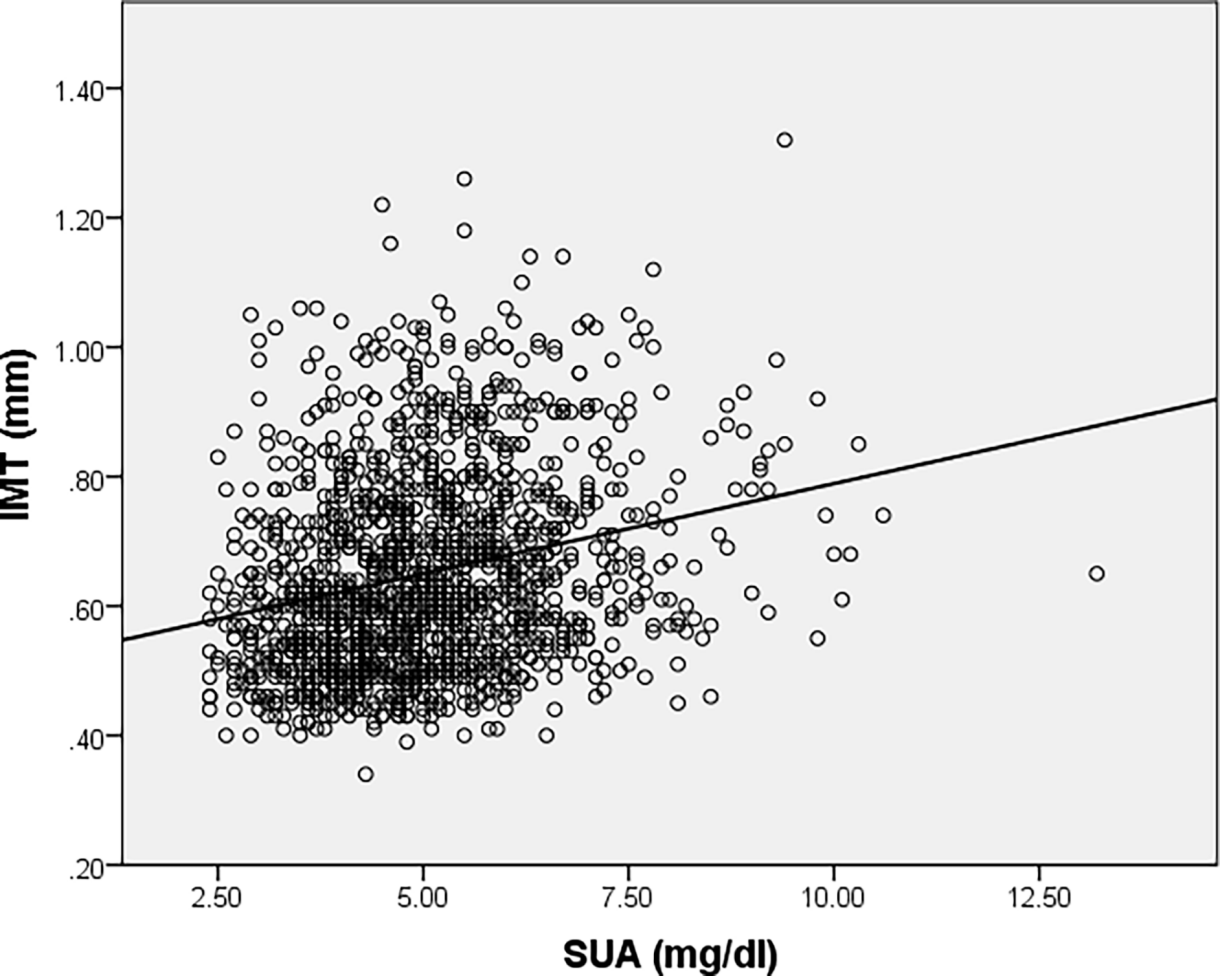
Correlation between serum uric acid levels and IMT values.

A partial correlation was considered to determine the relationship between an individual’s SUA level and IMT value whilst controlling for age. There was a low, direct correlation between SUA level (5.02 (1.379) mg/dL) and IMT value (0.65 (0.154) mm) whilst controlling for age (49.14 (17.596) years), which was statistically significant (r = 0.116, p<0.001, n = 1397). Data is summarized as mean (standard error). However, Sperman’s correlation coefficient showed that there was a statistically significant, low, direct correlation between SUA level and IMT value (r = 0.263, p<0.001, n = 1397), indicating that age had small influence in controlling for the relationship between SUA level and IMT value (Fig 2). To note that only a number of 1397 patients had a result of IMT values in our data.

**Fig 3:**
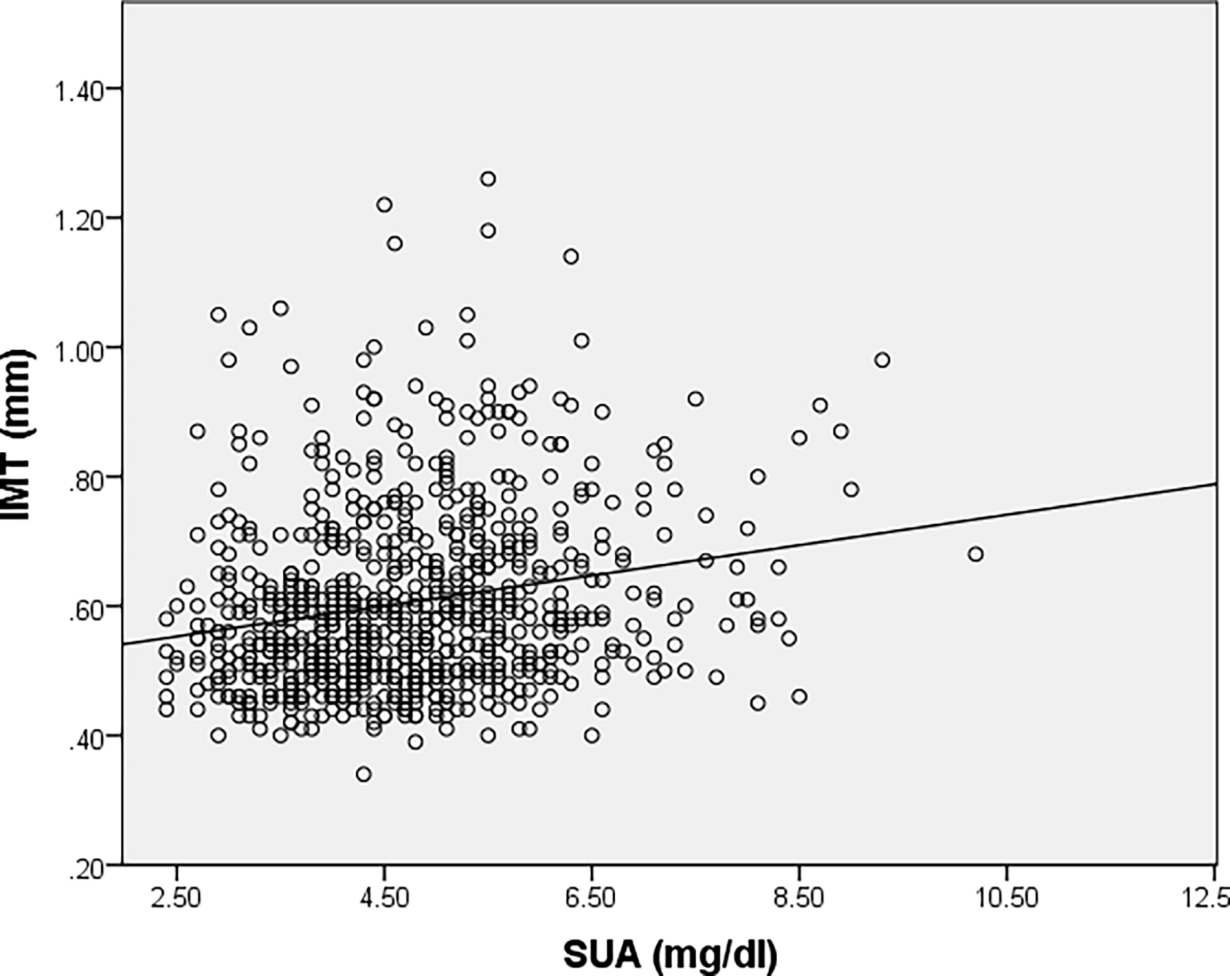
Correlation between serum uric acid levels and IMT values for subgroup of normotensive patients.

The subgroup of normotensive patients also presented a direct correlation between SUA levels and IMT values as revealed using Sperman’s correlation coefficient (r = 0.320, p<0.001) (Fig 3).

A significant difference in the mean value of GFR was obtained using both MDRD and CKD-EPI formulas. Hyperuricemia patients had a significant lower eGFR_MDRD_ levels, on average with 14.28ml/min/m2, compared to normouricemia patients (69.15 vs 83.22, p<0.001) respectively on average with 16.64, compared to normouricemia patients (74.05 vs 90.69, p<0.001) when using eGFR_CKD–EPI_.

The lower values of the estimated glomerular filtration rate (eGFR), assessed both by MDRD and by CKD-EPI formula, were observed in patients with hyperuricemia, these values being significantly lower than eGFR levels recorded in patients with normouricemia. All these differences remained statistically significant after adjusting by age, sex and BMI (Table 5). Values are summarized as mean (standard error).

**Table 5:** Serum uric acid levels and renal function: Estimated glomerular filtration rate (eGFR), assessed both by MDRD and by CKD-EPI formula, by groups of normouricemia and hyperuricemia patients; p-values are obtained with ANCOVA test.

	Normouricemia (N = 1694)	Hyperuricemia (N = 226)	p-value
**eGFR_MDRD_**			
**Unadjusted**	83.43 (0.448)	69.15 (1.227)	**<0.001**
**Adjusted for age**	82.83 (0.394)	73.67 (1.094)	**<0.001**
**Adjusted for gender**	83.43 (0.448)	69.16 (1.227)	**<0.001**
**Adjusted for BMI**	83.15 (0.443)	71.24 (1.241)	**<0.001**
**eGFR_CKD–EPI_**			
**Unadjusted**	90.69 (0.472)	74.05 (1.292)	**<0.001**
**Adjusted for age**	89.79 (0.347)	80.80 (0.963)	**<0.001**
**Adjusted for gender**	90.69 (0.470)	74.06 (1.288)	**<0.001**
**Adjusted for BMI**	90.29 (0.461)	77.05 (1.289)	**<0.001**

**Fig 4:**
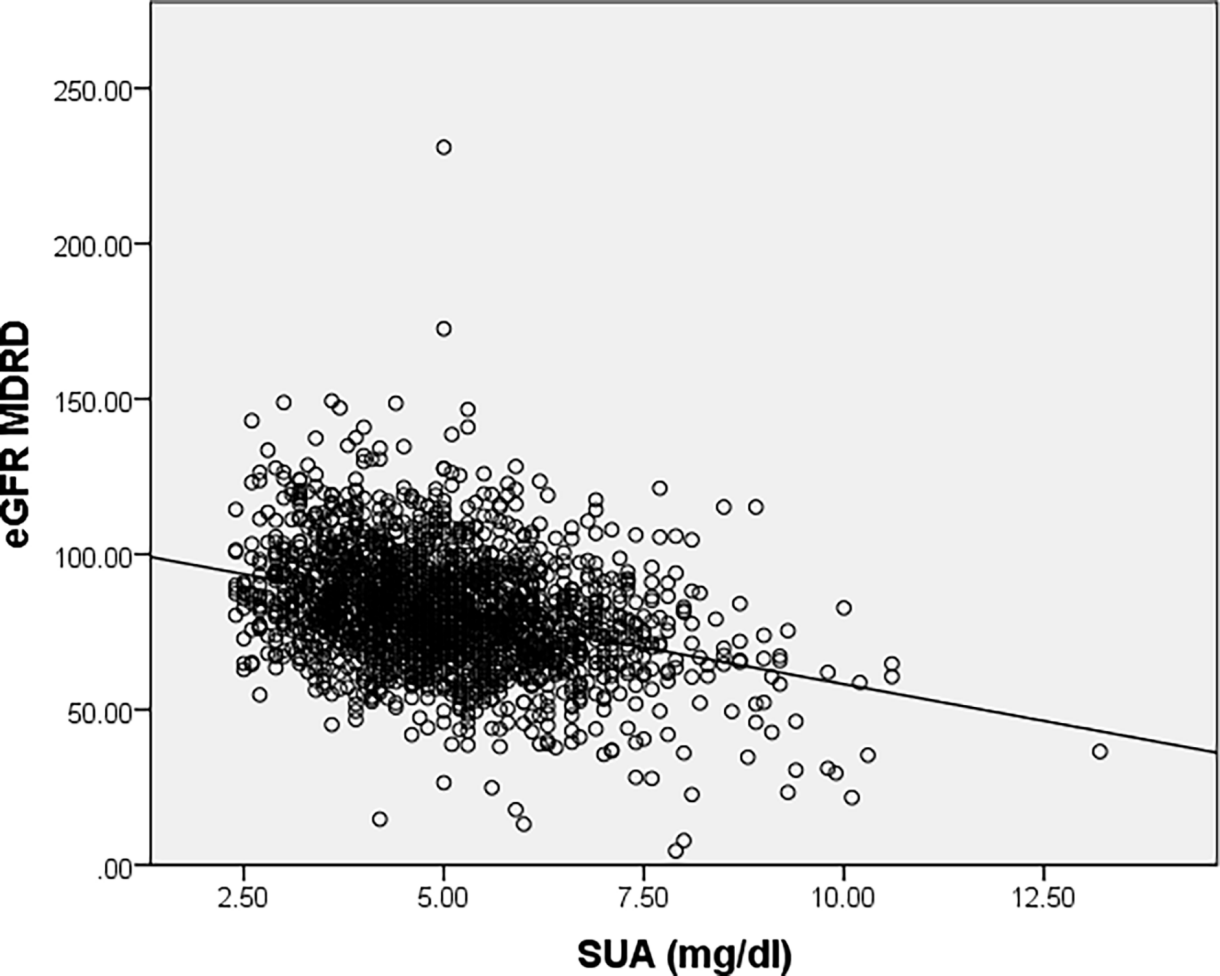
Correlation between serum uric acid levels and renal function assessed by eGFR_MDRD_.

**Fig 5:**
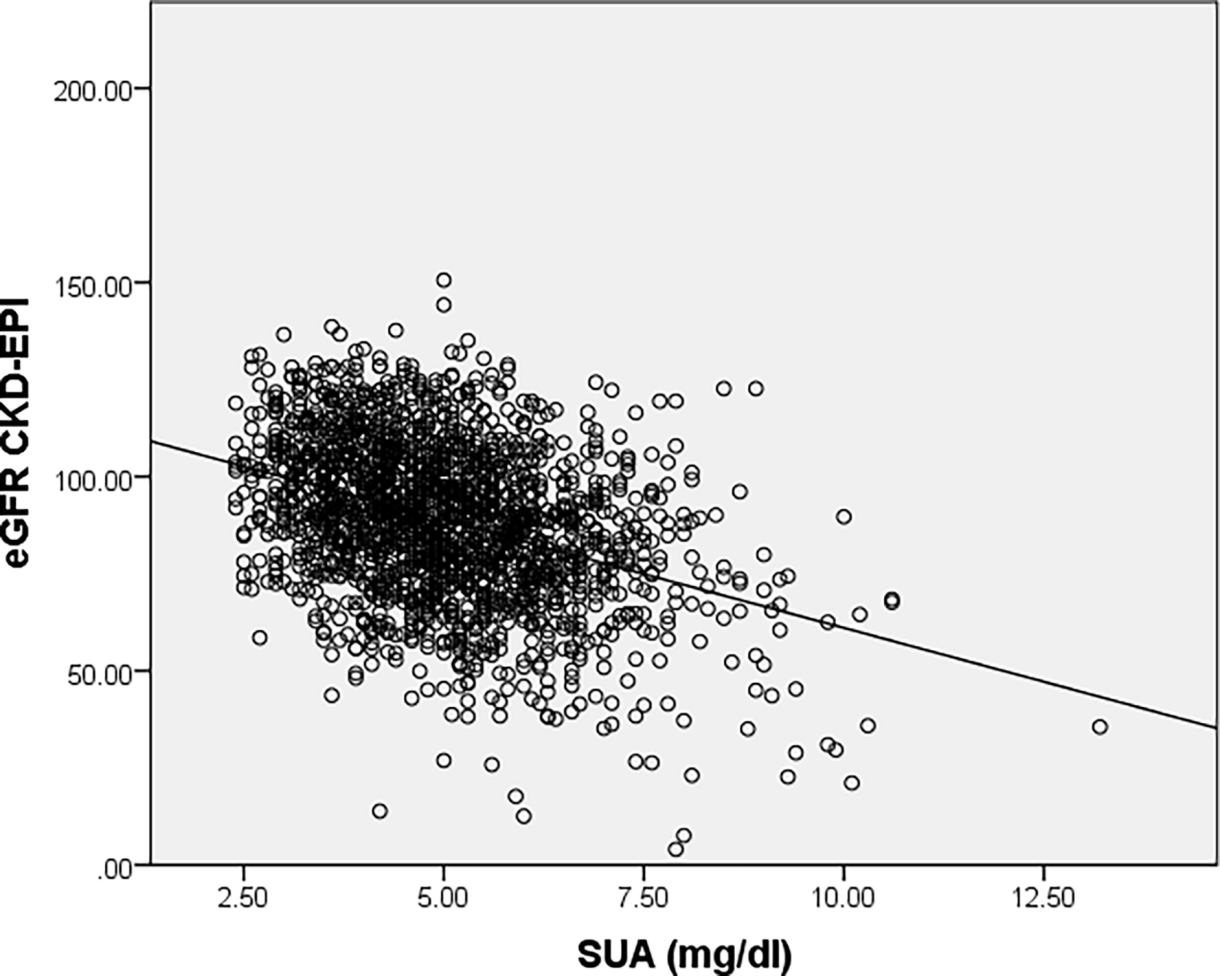
Correlation between serum uric acid levels and renal function assessed by eGFR_CKD–EPI_.

An indirect correlation between SUA levels and both eGFR levels was revealed by using Spearman’s correlation coefficient (r = -0.319, p<0.001) (Fig 4) and (r = -0.347, p<0.001) (Fig 5).

## Discussions

The same as for SEPHAR II survey, SEPHAR III data reconfirms SUA levels as an independent risk factor for HT development and negatively influence optimal BP control in treated HT subjects. This former issue is relevant to understanding whether elevated SUA levels impair the efficacy of BP lowering drugs and/or urate lowering therapy should be associated with antihypertensive drugs in these patients for reaching blood pressure control.

There have been several studies suggesting the strength of the relationship between serum UA and BP in different populations and ages [8] [9], our study being the first that provides specific data of Romanian population.

SEPHAR III results revealed the association between hyperuricemia in incident chronic kidney disease. The fact that eGFR values (evaluated both by MDRD and CKD-EPI) recorded among hyperuricemic subjects were significantly lower than the values recorded in normouricemic subjects, even after the adjustment for confounders, suggests that SUA levels could reflect in fact an early stage of renal impairment most likely through nephrosclerosis. These results are consistent with current evidence that supports the role of uric acid as marker and mediator of risk for progressive decline in renal function [10] [11]. In a recent review hypertension and chronic kidney disease showed concordant evidence in meta-analyses of observational studies and in some meta-analyses of randomized controlled trials but they were not statistically significant in Mendelian randomization studies [12; 13].

Also, the direct correlation between SUA levels and IMT values revealed by our analysis support the hypothesis that SUA may also be used as a surrogate marker of early atherosclerosis. Similar correlation was noticed between SUA levels and IMT considering normotensive patients (r = 0.320, p<0.001) and also hypertensive patients (r = 0.295, p<0.001), suggesting that hyperuricemia has a possible role in atherosclerosis development in both hypertensive and normotensive subjects and emphasizing its role as an independent CV risk factor and a chronic kidney disease predicting factor.

A recently published review on the SUA levels and health outcomes, based on the results from meta-analyses of observational studies, highlights suggestive evidence for the link between increased SUA levels and increased mortality from heart failure, hypertension, diabetes, chronic kidney disease and coronary heart disease [12]. Therefore in countries with high and very high CV mortality such as Romania, screening of SUA may be a useful tool to identify subjects with high CV risk who could benefit from a more active treatment evaluation in order to decrease theirs CV mortality.

Prospective studies are necessary to confirm these results that could also identify SUA levels cut-off values for defining categories of patients at risk for developing HT, chronic kidney disease or early atherosclerosis. Also, future prospective studies need to address the possible implications on CV mortality of the mass screening for hyperuricemia and of the use of urate lowering therapy in all hyperuricemic subjects.

## Conclusions

The results of our study offers support that increased SUA levels are associated with hypertension and the lack of optimal BP control in treated hypertensive subjects, with the decline in kidney function independent of age and with early stages of atherosclerosis evidenced by its association with increased IMT values. Therefore, our results suggest that mass screening for hyperuricemia may identify patients at risk of developing HT.

## Supporting information

S1 File Raw data supporting analysis. (XLSX)
